# Nectin-2 Acts as a Viral Entry Mediated Molecule That Binds to Human Herpesvirus 6B Glycoprotein B

**DOI:** 10.3390/v14010160

**Published:** 2022-01-16

**Authors:** Hirohito Ogawa, Daisuke Fujikura, Hikaru Namba, Nobuko Yamashita, Tomoyuki Honda, Masao Yamada

**Affiliations:** 1 Department of Virology, Okayama University Graduate School of Medicine, Dentistry and Pharmaceutical Sciences, 2-5-1 Shikata-cho, Kita-ku, Okayama 700-8558, Japan; hnamba@okayama-u.ac.jp (H.N.); noyamash@okayama-u.ac.jp (N.Y.); thonda@okayama-u.ac.jp (T.H.); 2 School of Veterinary Medicine, Kitasato University, Higashi 23-35-1, Towada 034-8628, Japan; fujikura@vmas.kitasato-u.ac.jp

**Keywords:** HHV-6B, nectin-2, CD112, CD134, virus entry, glycoprotein B

## Abstract

Human herpesvirus 6B (HHV-6B) is a T-lymphotropic virus and the etiological agent of exanthem subitum. HHV-6B is present in a latent or persistent form after primary infection and is produced in the salivary glands or transmitted to this organ. Infected individuals continue to secrete the virus in their saliva, which is thus considered a source for virus transmission. HHV-6B primarily propagates in T cells because its entry receptor, CD134, is mainly expressed by activated T cells. The virus then spreads to the host’s organs, including the salivary glands, nervous system, and liver. However, CD134 expression is not detected in these organs. Therefore, HHV-6B may be entering cells via a currently unidentified cell surface molecule, but the mechanisms for this have not yet been investigated. In this study, we investigated a CD134-independent virus entry mechanism in the parotid-derived cell line HSY. First, we confirmed viral infection in CD134-membrane unanchored HSY cells. We then determined that nectin cell adhesion molecule 2 (nectin-2) mediated virus entry and that HHV-6B-insensitive T-cells transduced with nectin-2 were transformed into virus-permissive cells. We also found that virus entry was significantly reduced in nectin-2 knockout parotid-derived cells. Furthermore, we showed that HHV-6B glycoprotein B (gB) interacted with the nectin-2 V-set domain. The results suggest that nectin-2 acts as an HHV-6B entry-mediated protein.

## 1. Introduction

Human herpesvirus 6 (HHV-6) is classified into two distinct species, HHV-6A and HHV-6B [1,2,3,4], and human herpesvirus 7 (HHV-7) [5]. It belongs to the genus *Roseolovirus* in the betaherpesvirus subfamily, which is a group of T-lymphotropic herpesviruses and the causal agents of exanthem subitum (ES), also known as roseola infantum. Roseoloviruses cause life-long infections, as older children and adults continue to intermittently shed the virus and virus DNA in their saliva [6].

Human CD134 (also known as OX40) has been identified as an entry receptor for HHV-6B [7]. This molecule is a member of the tumor necrosis factor receptor superfamily 4 (TNFRSF4) and is induced on CD4^+^ and CD8^+^ T cells [8]. The use of CD134 as an entry receptor is consistent with evidence that HHV-6B is a T-lymphotropic virus that propagates in primary activated T cells [9]. However, this virus was detected in the saliva, salivary glands [2,10,11], and livers of pediatric patients [12,13]. Furthermore, HHV-6B was also detected in brain autopsy tissues [14], and encephalopathy with ES has also been reported [15]. Notably, the expression of CD134 was not detected in the salivary glands, liver, or brain samples available from the Human Protein Atlas (http://www.proteinatlas.org) [16]. The tropism of HHV-6B for non-T cells that do not express CD134 might be analogous to the multiple receptor usage of various herpesviruses that target cells from different lineages [17,18].

During the entry of herpes simplex virus (HSV) type 1 (HSV-1), glycoprotein (g) D (gD) can interact with any of three classes of receptors: nectins [19,20], a tumor necrosis factor family member designated as herpesvirus entry mediator (HVEM) [21], or 3-*O*-sulfated heparan sulfate [22]. gB is required for infection, especially for efficient membrane fusion during virus entry. HSV-1 gB can interact with three distinct receptors: paired immunoglobulin-like type 2 receptor α (PILRα) [23], myelin-associated glycoprotein (MAG) [24], and non-muscle myosin II (NM-II) [17,25]. gD is a conserved glycoprotein among several alphaherpesviruses [26]. In general, there are three glycoproteins (gB, gH, and gL) which are thought to be essential for the entry of all herpesviruses [18]. For HHV-6A and HHV-6B, eight virion enveloped glycoproteins (gH, gL, gM, gN, gB, gO, gQ1, and gQ2) have been described [27,28,29,30]. The gH/gL/gQ1/gQ2 complex is a viral ligand for two HHV-6A- and HHV-6B-specific receptors, CD46 [31] and CD134 [7], respectively. Another trimetric complex (gH/gL/gO) [32] and gB are also candidate virus entry factors; however, cellular receptors that bind to these glycoproteins have not yet been discovered. One hypothesis is that a novel cellular receptor may mediate HHV-6B entry into cells.

The frequent detection of HHV-6 DNA, especially HHV-6B DNA in saliva and salivary glands [2,11,33], suggests that the salivary glands are a potential site of HHV-6 persistence [34]. Therefore, it is important to elucidate how HHV-6B infects salivary glands that do not express CD134. To address this, we identified an unknown cell surface molecule using a full-length cDNA library prepared from the parotid gland cell line, HSY [35]. We found that nectin cell adhesion molecule 2 (nectin-2) [36], previously named HveB and also known as CD112, mediated the entry of HHV-6B. gB acted as a ligand for nectin-2, and there was a binding region for entry in the V-set domain. Our results suggest that nectin-2 acts as an HHV-6B entry-mediated molecule.

## 2. Materials and Methods

### 2.1. Cells and Virus Strains

The T-cell lines MT-4, Sup-T1, and CCRF-HSB-2 and their transfectants were cultured in Roswell Park Memorial Institute (RPMI) 1640 medium supplemented with 100 U/mL penicillin, 100 mg/mL streptomycin (Sigma-Aldrich, St. Louis, MO, USA), and 10% fetal bovine serum (FBS). The parotid gland cell line (HSY and its transfectants) [35], 293T cells, and Platinum-GP retroviral packaging (Plat-GP) cells used for the production of retrovirus vectors (Cell Biolabs, San Diego, CA, USA) were all cultured in Dulbecco’s modified Eagle’s minimal essential medium (DMEM) supplemented with 100 U/mL penicillin, 100 mg/mL streptomycin (Sigma-Aldrich), and 10% FBS. HHV-6B strains HST and Z29 were propagated on cord blood mononuclear cells, and virus stocks were prepared as described previously [37].

### 2.2. Reverse Transcription-PCR (RT-PCR) and Quantitative RT-PCR

Total RNA was extracted using a NucleoSpin RNA Plus kit (Macherey-Nagel, Düren, Germany) according to the manufacturer’s instructions. One-step RT-PCR was carried out using a Qiagen OneStep RT-PCR kit (Qiagen, Hilden, Germany) according to the manufacturer’s instructions. HHV-6B U91 and U100 genes were amplified using the following primer sets: 5′-GGCGCTGAAGCATGTAAGCA-3′ and 5′-CAGAACAGTCAGGTATTTTCCCC-3′ for U91 [38] and HHV6B-U100-mRNA-F (5′-TGCACCATGATCGTCCTACG-3′) and HHV6B-U100-mRNA-R (5′-TCGCATTCCGATGGTTTCCA-3′) for U100, which were designed in this study. The β-actin gene (*ACTB*) was amplified using hACTB-mRNA71F (5′-GCCGCCAGCTCACCAT-3′) and hACTB-mRNA299R (5′-TCGATGGGGTACTTCAGGGT-3′), which were designed in this study.

For the quantitative RT-PCR of *NECTIN2*, total RNA was subjected to cDNA synthesis using a PrimeScript II 1st strand cDNA Synthesis Kit (Takara Bio, Shiga, Japan), and 10 ng cDNA was amplified using a TB Green *Premix Ex Taq* II kit (Takara Bio) and an Applied Biosystems StepOnePlus System (Thermo Fischer Scientific, Waltham, MA, USA), all according to the manufacturer’s instructions. *NECTIN2* isoforms were amplified using the following primer sets: NEC2-alpha-qF (5′-TCTACGATCCGAAAGCTCAGGTGT-3′) and NEC2-alpha-qR (5′-CATCCTTGCCATCTGGTTCCATGG-3′) for *NECTIN2* alpha and NEC2-delta-qF (5′-TCGGAGCACAGCCCACTCAAGAC-3′) and NEC2-delta-qR (5′-GTGGGCAGCTCATGGTATCGAGG-3′) for *NECTIN2* delta, which were designed in this study.

### 2.3. Antibodies

APC anti-human CD134 monoclonal antibody (mAb) (clone: Ber-ACT35), APC mouse immunoglobulin (Ig) G 1, κ isotype control mAb (clone: MOPC-21), PE anti-human CD112 (Nectin-2) mAb (clone: TX31), and PE mouse IgG1, κ isotype control mAb (clone: MOPC-21) were purchased from BioLegend (San Diego, CA, USA). Anti-HHV-6A gQ1 mAb (clone: 119) was purchased from Cosmo Bio (Tokyo, Japan). Anti-nectin-2 mAb (clone: E-1), anti-HHV-6 gB (clone: 6A5), and anti-HHV-6 p41 early antigen mAb (clone: 9A5D12) were purchased from Santa Cruz Biotechnology (Dallas, TX, USA). Mouse IgG 2B isotype control mAb (clone: 20116) were purchased from Bio-techne (Minneapolis, MN, USA). Anti-DDDDK-tag mAb (clone: FLA-1) and anti-hemagglutinin (HA)-tag mAb (clone: TANA2) were purchased from MBL (Nagoya, Japan). Anti-β-tubulin mAb was purchased from Fujifilm Wako (Osaka, Japan). Alexa Fluor 488 conjugated goat anti-mouse IgG (H+L) F(ab’)2 (A-11017) and Alexa Fluor 555 conjugated goat anti-mouse IgG (H+L) F(ab’)2 (A-21425) were purchased from Thermo Fisher Scientific. Horseradish peroxidase (HRP)-conjugated goat anti-mouse IgG (H+L) was purchased from Jackson ImmunoResearch (115-035-062; West Grove, PA, USA). HRP-conjugated anti-mouse IgG for immunoprecipitation (IP) was purchased from Abcam (ab131368; Cambridge, UK).

### 2.4. Flow Cytometric Analysis

Cells were washed with cold fluorescence-activated cell sorting (FACS) buffer (phosphate-buffered saline (PBS) containing 1% sodium azide and 2% FBS) and incubated with the indicated mAbs at 4 °C for 30 min, after blocking with purified mouse IgG (BioLegend, clone MG1-45, 0.5 μg/mL, 4 °C, 15 min). After several washes, the cells were stained with 7-AAD Viability Staining Solution (BioLegend) and analyzed using a BD FACSAria III Cell Sorter (BD, Franklin Lakes, NJ, USA) and FlowJo software version 10 (BD).

### 2.5. Immunofluorescence Assay (IFA)

HSY cells and ex6 C7 cells were grown on poly L-lysine-coated micro slide glasses (Matsunami Glass, Osaka, Japan). Cells were infected with HHV-6B and cultured for 2–4 days. T-cell lines (MT-4, CCRF-HSB-2, and its transformants) were infected with the virus and cultured for 1–7 days in 24-well plates (AS ONE, Osaka, Japan). After centrifugation, the cells were transferred to 14-well ring marked micro slide glasses (Matsunami Glass) and air-dried. After fixation with cold acetone for 5 min, the cells were incubated with primary mAb for 25 min at 35 °C. After washing, the bound antibodies were detected with secondary fluorescent dye-conjugated antibodies for 25 min at 35 °C. After several washes, cover slides were mounted using ProLong Diamond Antifade Mountant with DAPI (Thermo Fisher Scientific). Fluorescent images were acquired using a BZ-X700 microscope (Keyence, Osaka, Japan).

### 2.6. Establishment of a CD134-Membrane Unanchored HSY Cell Clone (Ex6 C7)

The transmembrane region of *TNFRSF4* was mutated using the CRISPR/Cas9 system, as described previously [39]. The 23-nucleotide guide sequence (5′-CCGTGCGGTTGCCGCCATCCTGG-3′) targeting the negative strand genomic DNA located on exon-6 of the human *TNFRSF4* gene was designed using a CRISPR direct software uploaded to http://crispr.dbcls.jp. The DNA fragment was cloned into pSpCas9(BB)-2A-Puro (PX459) ver.2 [40]. The resulting plasmid was transfected into HSY cells using Lipofectamine 2000 (Thermo Fisher Scientific). Two days after puromycin selection at 0.125 μg/mL, single-cell clones were isolated using a limiting dilution. Several cell clones were selected, and their genomic DNA was sequenced to confirm gene mutations. The transmembrane helices were predicted using TMHMM2.0 [41]. One clone (ex6 C7 cells) was established.

### 2.7. Establishment of Nectin-2 Knockout Ex6 C7 (Ex6 C7 Nec2-KO) Cell Clones

The *NECTIN2* gene was mutated using the CRISPR/Cas9 system, as described above. Briefly, the 23-nucleotide guide sequence (5′-CCTGCCGTCGAGATCGCCGCCGA-3′) targeting the negative strand genomic DNA of the human *NECTIN2* gene was designed and cloned into a plasmid vector. The resulting plasmid was transfected into ex6 C7 cells, and the obtained cell clones were sequenced to confirm the gene mutations (Figure S1). Three clones (ex6 C7 nec2-KO1, -KO2, and -KO3) were established.

### 2.8. Expression Screening

A full-length cDNA library was constructed from ex6 C7 cells using the In-Fusion SMARTer Directional cDNA Library construction kit (Takara Bio) according to the manufacturer’s instructions and cloned into a Molony murine leukemia virus (MMLV)-based retroviral vector, pMX (Cell Biolabs), as described previously [39,42]. The titer of the cDNA library was more than 1 × 10^6^ colony-forming units, and more than 80% of the individual clones contained different cDNA fragments. Plat-GP cells were co-transfected with pMX containing cDNA library genes and a VSV G-expressing plasmid, pCMV-VSV-G (Cell Biolabs), using Lipofectamine 2000. Two days later, culture supernatants were collected and cleared of cell debris by centrifugation at 300× *g* for 5 min and then passed through 0.45 μm pore filters (Merck Millipore, Darmstadt, Germany). CCRF-HSB-2 cells (5 × 10^5^ cells) were infected with viruses at a multiplicity of infection of 0.1 with 0.8 μg/mL polybrene solution (Nacalai Tesque, Kyoto, Japan). Cells (lib-CCRF-HSB-2 cells) were expanded for several passages and stored at −80 °C. The lib-CCRF-HSB-2 cells (1.2 × 10^5^ cells) were infected with HHV-6B and cultured for several passages to generate HHV-6B persistently infected lib-CCRF-HSB-2 cells. The cells were incubated with anti-HHV-6A gQ1 mAb for 30 min at 4 °C. After two washes with cold PBS, the cells were stained with anti-mouse IgG-Alexa Fluor 488 and APC anti-human CD134 for 30 min at 4 °C. After two washes, the cells were separated on a BD FACSAria III by dot plots gating on Alexa Flour 488 (gQ1) versus APC (CD134). Both Alexa Fluor 488-positive and APC-negative cells were considered HHV-6B-infected cells (non CD134-dependent) and sorted. Then, the sorted cells were expanded. After several rounds of sorting and culturing, genomic DNA was extracted using a QIAamp DNA Mini Kit (Qiagen) according to the manufacturer’s instructions, and library genes inserted into the genomic DNA were amplified by PCR using pMXs-specific primers and then sequenced.

### 2.9. Generation of Nectin-2- and CD134-Expressing Cell Lines

Total RNA was extracted from HSY cells using the NucleoSpin RNA Plus kit (Macherey-Nagel), and mRNA was reverse transcribed using a PrimeScript II 1st strand cDNA Synthesis Kit (Takara Bio), all according to the manufacturer’s instructions. The *NECTIN2* (isoform δ) and *TNFRSF4* genes were amplified from cDNA with KOD One (Toyobo, Osaka, Japan) and inserted into pMXs-IRES-GFP (Cell Biolabs). After sequence confirmation, the plasmids were retrovirally transfected into CCRF-HSB-2 cells as described above. Transduced GFP-positive cells were collected using a BD FACSAria III Cell Sorter (BD Biosciences) and then used in the experiments (HSB2-nec2δ, HSB2-CD134, and HSB2-cont cells).

### 2.10. Generation of Nectin-2- and CD134-Expressing Cell Lines

Plasmids encoding FLAG-tagged nectin-2 (pCAGGS-nec2δ-FLAG) were prepared. Briefly, the *NECTIN2*-δ gene was amplified from cDNA as prepared above using KOD One (Toyobo) and inserted into pCAGGS [43] with in vivo *Escherichia coli* cloning (iVEC) technology [44]. After sequence confirmation, the plasmids of the mutant nectin-2 were constructed using a PrimeSTAR Mutagenesis Basal Kit (Takara Bio) and ECOS Competent *E. coli* DH5α (Nippon Gene, Tokyo, Japan), all according to the manufacturer’s instructions. The resulting plasmids were designated pCAGGS-nec2δ-ΔV-FLAG (mutant lacking nectin-2δ V-set domain), pCAGGS-nec2δ-ΔC2×2-FLAG (mutant lacking nectin-2δ C2-set domains), pCAGGS-nec2δ-ΔC2(55aa)-FLAG (mutant lacking nectin-2δ first C2-set domain), and pCAGGS-nec2δ-ΔC2(46aa)-FLAG (mutant lacking nectin-2δ second C2-set domain).

Plasmids encoding HA-tagged HHV-6B gB (pCAGGS-HST gB-HA and pCAGGS-Z29 gB-HA) were prepared as follows: Total RNA was extracted from HHV-6B HST and Z29 strains using the NucleoSpin RNA Plus kit (Macherey-Nagel) and mRNA was reverse transcribed using a PrimeScript II 1st strand cDNA Synthesis Kit (Takara Bio), all according to the manufacturer’s instructions. The gB genes were amplified from cDNA using KOD One (Toyobo) and inserted into pCAGGS using an In-Fusion HD Cloning Kit (Takara Bio) according to the manufacturer’s instructions. The plasmids were transformed into MAX Efficiency Stbl2 competent cells (Thermo Fischer Scientific), and the transformants were cultured at 30 °C. After sequence confirmation, the mutant HST gB-HA plasmid lacking a transmembrane domain was constructed using a PrimeSTAR Mutagenesis Basal Kit (Takara Bio) and MAX Efficiency Stbl2 competent cells (Thermo Fischer Scientific), all according to the manufacturer’s instructions.

A plasmid encoding HA-tagged HST gQ1 (pCAGGS-HST gQ1-HA) was prepared as described above. Empty vector control plasmids (pCAGGS-FLAG and pCAGGS-HA) were also constructed as controls.

### 2.11. Transfection, Immunoblotting, and Co-IP

The 293T cells were grown in 6-well plates 24 h before transfection at 37 °C and 5% CO_2_. The plasmids were transfected into cells using polyethylenimine “Max” (1 mg/mL) (Polysciences, Warrington, PA, USA). At 72 h post-transfection, cells were washed four times with cold PBS, and then treated with 0.25 mL of cold lysis buffer (20 mM Tris-HCl (pH 7.4), 135 mM NaCl, 1% Triton X-100, 1% glycerol) containing a protease inhibitor cocktail (25955-11; Nacalai Tesque) for 15 min at 4 °C. Cell lysates were sonicated at 4 °C for 10 s and centrifuged at 21,000× *g* for 10 min at 4 °C. Aliquots of the supernatants were collected and stored at −80 °C until the immunoblotting. The remaining supernatants were mixed with anti-DDDDK-tag mAb-magnetic agarose (M185-10; MBL) or anti-HA-tag mAb-magnetic agarose (M132-10; MBL) and incubated on a rotator at 4 °C overnight. After washing four times with cold lysis buffer, the beads were boiled with 30 μL of sodium dodecyl sulfate (SDS) sample buffer and subjected to 10% SDS-polyacrylamide gel electrophoresis. The proteins were transferred to polyvinylidene fluoride membranes (Immobilon-P; Merck Millipore), which were blocked with 3% skim milk in PBS containing 0.05% Tween 20 for 1 h at room temperature, followed by incubation with the appropriate primary antibodies for 1 h at room temperature. After washing, the bound antibodies were detected using HRP-conjugated secondary antibodies. The bound antibodies were visualized with Immobilon Western (Merck Millipore) and detected using a C-DiGit Blot Scanner (LI-COR Biosciences, Lincoln, NE, USA). The experiment was performed at least thrice, and representative data are shown.

### 2.12. Nectin-2 Binding Assay in HHV-6B-Infected Cells

MT-4 cells were infected with the virus (multiplicity of infection = 0.1) and cultured for 6 d. Cells were washed with cold PBS and then treated with cold lysis buffer (0.5 mL) containing a protease inhibitor cocktail (Nacalai Tesque) for 15 min at 4 °C. Cell lysates were sonicated at 4 °C for 10 s and centrifuged at 21,000× *g* for 10 min at 4 °C. The supernatants were incubated on a rotator at 4 °C for 4 h and mixed with anti-HHV-6 gB antibody (6A5) binding Dynabeads Protein A (Thermo Fisher Scientific) on a rotator at 4 °C overnight. After washing four times with cold lysis buffer, the beads were boiled with 30 μL of SDS sample buffer and subjected to immunoblotting. The experiment was performed at least thrice, and representative data are shown.

### 2.13. Assay of Nectin-2 Binding to Purified HHV-6B gB

A plasmid expressing soluble HST gB-HA protein was transfected into 293T cells grown in 6-well plates, as described above. At 72 h post-transfection, the collected soluble gB-HA proteins were mixed with anti-HA-tag mAb-magnetic agarose (MBL) and incubated on a rotator at 4 °C for 1 h. The proteins were purified by washing four times with cold lysis buffer and suspended in 0.4 mL of cold lysis buffer. The purified gB-HA binding magnetic agarose was incubated with 0.4 μg of human CD112 Fc chimera recombinant protein (A42495; Thermo Fisher Scientific) or recombinant human IgG1 Fc (778304; BioLegend) on a rotator at 4 °C for 1 h. After washing four times with cold lysis buffer, the magnetic agaroses were boiled with 30 μL of SDS sample buffer and subjected to immunoblotting. The experiment was performed twice, and representative data are shown.

### 2.14. Statistical Analysis

Statistical analyses were performed using GraphPad PRISM software version 8 (GraphPad Software, San Diego, CA, USA).

## 3. Results

### 3.1. HHV-6B Infects the HSY Parotid Gland Cell Line

**Figure 1:**
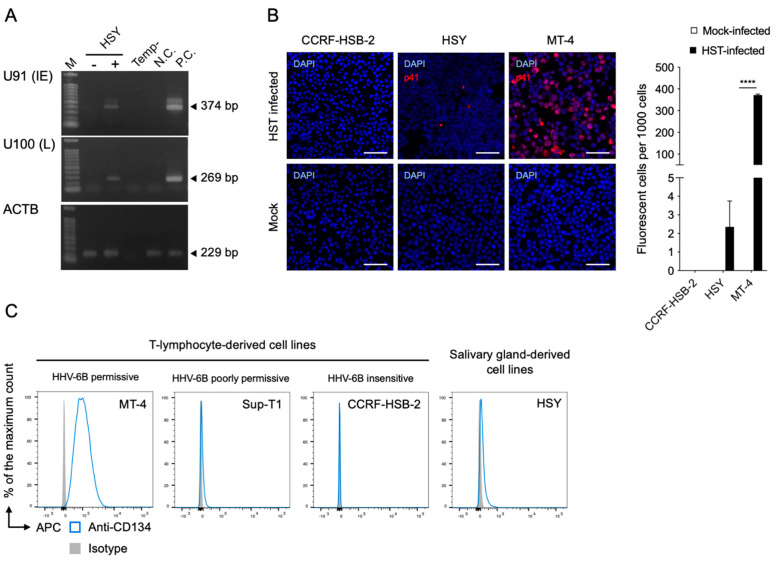
HHV-6B infectivity in HSY cells and relative CD134 expression. (**A**) Viral RNA detection in HSY cells using RT-PCR. Cells were infected with HHV-6B HST and total RNA was extracted at 48 h post-infection. U91 (immediate early) and U100 (late) transcripts were detected. β-actin (*ACTB*) was used as an internal control. M, DNA ladder; -, mock-infected HSY cells; +, HST-infected HSY cells; Temp-, No template control; N.C., negative control (HST-infected CCRF-HSB-2 cells); P.C., positive control (HST-infected MT-4 cells). (**B**) Viral antigen detection in HSY cells using an immunofluorescence assay. Cells were infected with HHV-6B HST and treated with cold acetone at 48 h post-infection (left panel). HHV-6 p41 protein (red) was detected. Nuclei of cells were stained with DAPI (blue). Scale bars represent 50 μm. Virus-infected cells (red) were counted (right panel). Error bars represent standard deviations (*n* = 3 per cell). Asterisks indicate statistical significance (**** *p* < 0.0001, Bonferroni’s multiple comparison test). (**C**) CD134 expression on T-lymphocyte cell lines and HSY cells was analyzed using fluorescence-activated cell sorting. Cells were stained with APC-conjugated anti-CD134 or isotype control antibodies. The antibody specific signals of the cells are shown in the histogram.

To determine whether HHV-6B infects salivary gland derivative cells, we infected HSY cells with an HHV-6B HST strain and detected the viral RNA using RT-PCR. U91 (immediate early) and U100 (late) mRNAs were detected in the HSY cells (Figure 1A). The viral antigen was also detected in HSY cells by IFA using an anti-HHV-6 p41 early antigen antibody (Figure 1B). We then compared the expression levels of the CD134 in the HSY and T-lymphocyte cell lines, which have a different HHV-6B-permissive range, using FACS analysis. The CD134 was highly and slightly expressed in the HHV-6B-permissive T-cell line (MT-4) and HHV-6B-poorly permissive T-cell line (Sup-T1), respectively (Figure 1C). CD134 was not detected in the HHV-6B-insensitive T-cell line (CCRF-HSB-2). CD134 was detected only slightly in the HSY cells (Figure 1C).

### 3.2. HHV-6B Infects Ex6 C7 Cells (CD134-Membrane Unanchored HSY Cells)

**Figure 2:**
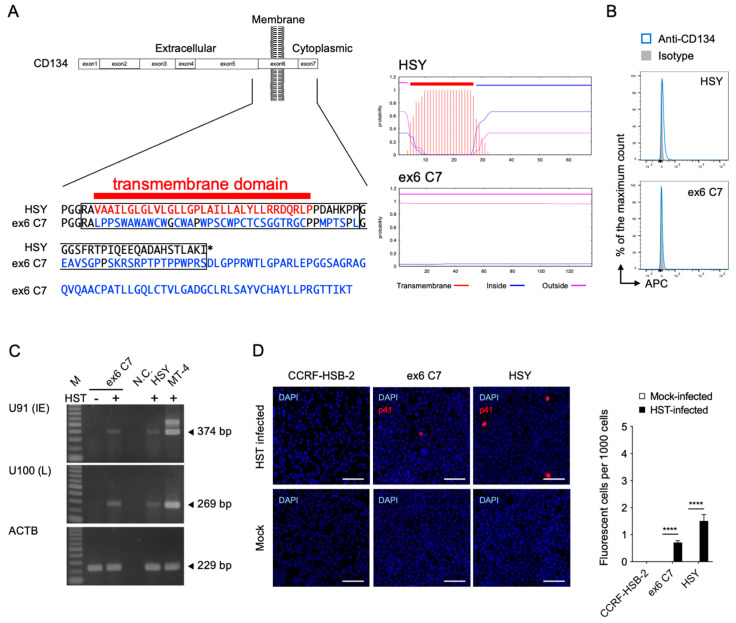
Establishment of *TNFRSF4* gene-specific mutated HSY cells (ex6 C7 cells) and HHV-6B permissivity. (**A**) Comparison of amino acid sequences and predicted transmembrane helices around the target region in TMHMM2.0 [41]. In ex6 C7 cells, a frameshift mutation near the *N*-terminus of the CD134 transmembrane region (left panel) led to translation of an amino acid sequence not capable of serving as a transmembrane region (right panels). (**B**) CD134 expression by HSY cells and ex6 C7 cells was analyzed by means of fluorescence-activated cell sorting analysis. (**C**) Viral RNA detection in ex6 C7 cells by RT-PCR. Cells were infected with HHV-6B HST and total RNA was extracted at 48 h post-infection. U91 (immediate early) and U100 (late) transcripts were detected. β-actin (ACTB) was used as an internal control. M, DNA ladder; -, mock-infected cells; +, HST-infected cells; N.C., no template control. (**D**) Viral antigen detection in ex6 C7 cells and HSY cells using immunofluorescence assay. Cells were infected with HHV-6B HST and treated with cold acetone at 48 h post-infection (left panel). HHV-6 p41 protein (red) was detected in both cell types. Nuclei of cells were stained with DAPI (blue). Scale bars represent 50 μm. Virus-infected cells (red) were counted (right panel). Error bars represent standard deviations (*n* = 3 per cell). Asterisks indicate statistical significance (**** *p* < 0.0001, Bonferroni’s multiple comparison test).

To determine the requirement of CD134 for viral entry into the HSY cells, a *TNFRSF4* gene-specific mutation was performed using the CRISPR-Cas9 system [40]. Because the *TNFRSF4* gene has several transcript variants, it was difficult to establish a complete CD134 knockout cell clone. Instead, the transmembrane region of the *TNFRSF4* gene was deleted so that CD134 was expressed in the HSY cells without its transmembrane anchor sequence. Examination of the resulting amino acid sequence showed that a native transmembrane segment had been lost and replaced with amino acids that were not predicted to form a hydrophobic transmembrane anchor (Figure 2A). As expected, we obtained ex6 C7 cells lacking CD134 expression on their surfaces (Figure 2B). The mutant CD134 did not appear to be present inside the cells (Figure S2). Interestingly, we detected U91 and U100 mRNA by RT-PCR as well as p41 early antigen by IFA in the HST strain-inoculated ex6 C7 cells, although CD134 was not detected on the cell surface (Figure 2C,D). These data suggest that HHV-6B enters ex6 C7 cells using an unknown cell surface molecule that is not CD134.

### 3.3. Nectin-2 Is Required for HHV-6B Infection

To identify the potential receptor molecules required for HHV-6B entry, we constructed a cDNA library obtained from ex6 C7 cells in a retroviral vector, pMX, and retrovirally transfected the cDNA library into an HHV-6B insensitive T-cell line, CCRF-HSB-2 (lib-CCRF-HSB-2). We inoculated HHV-6B into lib-CCRF-HSB-2 cells, and then isolated the transfectants detected as gQ1-positive via cell sorting. After several rounds, a *NECTIN2* fragment was identified. Nectin-2 is a Ca^2+^-independent Ig-like cell adhesion molecule (CAM) belonging to the Ig superfamily and is ubiquitously expressed in a variety of cells [36,45]. Nectin-2 is an entry receptor for alphaherpesviruses, HSV-1 mutant strains, HSV-2, and pseudorabies virus (PRV) and interacts with gD [20,46,47].

**Figure 3:**
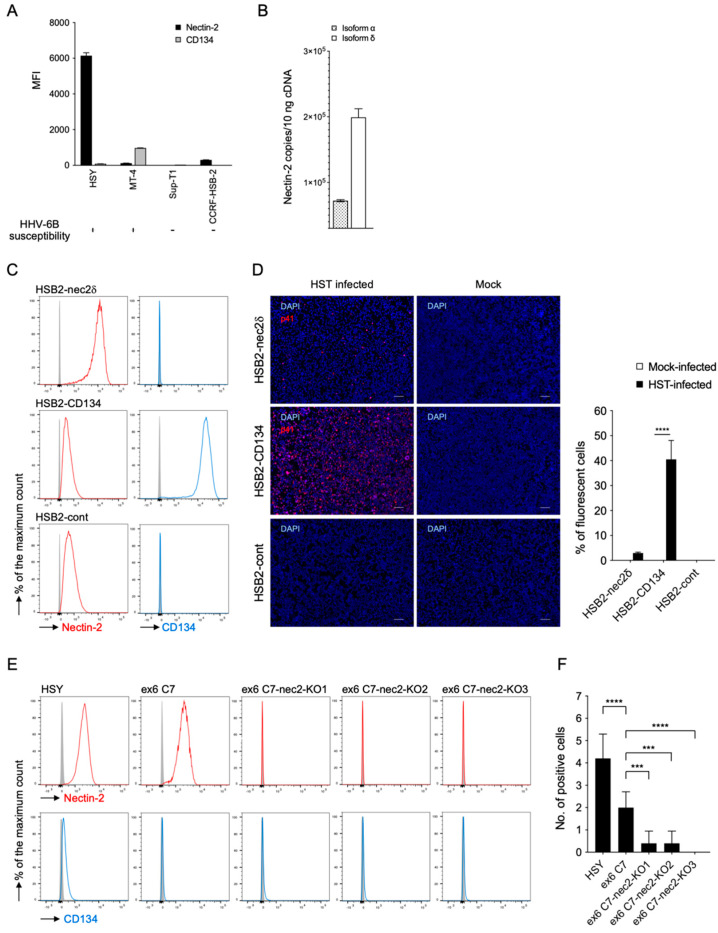
Nectin-2 is expressed on HSY cells, and HHV-6B-infected cells express different levels of nectin-2. (**A**) Median fluorescence intensity (MFI) of nectin-2 expressed on the cell surface. HSY cells had a greater intensity of nectin-2 staining when compared with the T-cell lines, MT-4, Sup-T1, and CCRF-HSB-2 cells. Error bars represent standard deviations (*n* = 3 per cell). (**B**) Mean copy numbers of nectin-2 isoforms-α and -δ mRNA. Isoform δ was dominantly detected in HSY cells. Error bars represent standard deviations (*n* = 3 per cell). (**C**) Nectin-2 and CD134 expression levels in MMLV-transduced cells and parent CCRF-HSB-2 cells. (**D**) Cells were infected with HHV-6B HST and treated with cold acetone at 96 h post-infection (left panel). HHV-6 p41 protein (red) was detected in HSB2-nec2δ cells and HSB2-CD134 cells with an immunofluorescence assay. Nuclei of cells were stained with DAPI (blue). Scale bars represent 50 μm. Virus-infected cells (red) were counted (right panel). Error bars represent standard deviations (*n* = 3 per cell). Asterisks indicate statistical significance (**** *p* < 0.001, Bonferroni’s multiple comparison test). (**E**) Nectin-2 and CD134 expression levels in HSY cells, ex6 C7 cells, and three lines of *NECTIN2* gene-specific mutated ex6 C7 cells (ex6 C7-nec2-KO cells) were detected using FACS. (**F**) HSY cells, ex6 C7 cells, and ex6 C7-nec2-KO cells were inoculated with the HHV-6B HST strain. HHV-6 p41 protein (red) was detected at 48 h post-infection by IFA. Positive cells were determined by counting the number of red signals in a microscopic field. Error bars represent standard deviations (*n* = 5 per cell). Asterisks indicate statistical significance when compared with the parent ex6 C7 cells (*** *p* < 0.001, **** *p* < 0.0001, Dunnett’s multiple comparison test).

To assess the expression level of nectin-2, we performed FACS analyses in HSY cells, which demonstrated that they had a greater intensity of nectin-2 staining than other T-cell lines (Figure 3A). This molecule has two splicing variants, nectin-2α (short form) and -2δ (long form), which share an ectodomain and differ in the transmembrane and cytoplasmic regions [36]. Nectin-2δ, but not nectin-2α, is tyrosine phosphorylated in response to cell-to-cell adhesion [48]. To confirm which isoform was dominant in HSY cells, total RNA was extracted from the cells, and nectin-2α and-2δ mRNA levels were determined using quantitative RT-PCR. As shown in Figure 3B, larger amounts of nectin-2δ mRNA were detected than nectin-2α mRNA, indicating that nectin-2δ was the dominant type. Therefore, the subsequent results are mainly related to nectin-2δ.

Next, we generated CCRF-HSB-2 cells that overexpressed nectin-2δ (HSB2-nec2δ cells) (Figure 3C). We also generated CCRF-HSB-2 cells overexpressing CD134 (HSB2-CD134 cells) and CCRF-HSB-2 cells into which the empty plasmid was retrovirally transfected (HSB2-cont cells), and then analyzed their susceptibility to HHV-6B infection (Figure 3C,D). Interestingly, HSB2-nec2δ cells were susceptible to viral entry; but the rate was much lower than that of the HSB2-CD134 cells. While the HSB2-cont cells expressed nectin-2 and it was detectable by FACS, they did not permit viral replication.

To evaluate the importance of endogenous nectin-2 in HSY cells for HHV-6B, we generated nectin-2-knockout ex6 C7 (ex6 C7-nec2-KO) cells using the CRISPR-Cas9 system. Although HHV-6B infection inhibition assays using soluble nectin-2 or anti-nectin-2 antibodies are standard, the baseline situation is that the efficiency of HHV-6B is low in HSY cells (Figure 1, Figure 2 and Figure 3); this makes it difficult to show the difference between the presence and absence of soluble nectin-2 and the antibody. Alternatively, we selected a method to generate ex6 C7-nec2-KO cells and examined viral infection. We obtained three cell clones (ex6 C7-nec2-KO1, -KO2, and -KO3) that were confirmed to lack nectin-2 expression by FACS (Figure 3E). These cells were inoculated with the HHV-6B HST strain, and their susceptibility to HHV-6B infection was analyzed by IFA. Interestingly, viral antigen-positive cells were significantly reduced in all nectin-2-knockout ex6 C7 cells when compared with the parent ex6 C7 cells (Figure 3F).

These data suggest that nectin-2 is a viral entry-mediated molecule involved in the infection of salivary gland cells by HHV-6B.

### 3.4. Nectin-2 Is a Functional Entry-Mediated Molecule for HHV-6B HST and Z29 Strains

**Figure 4:**
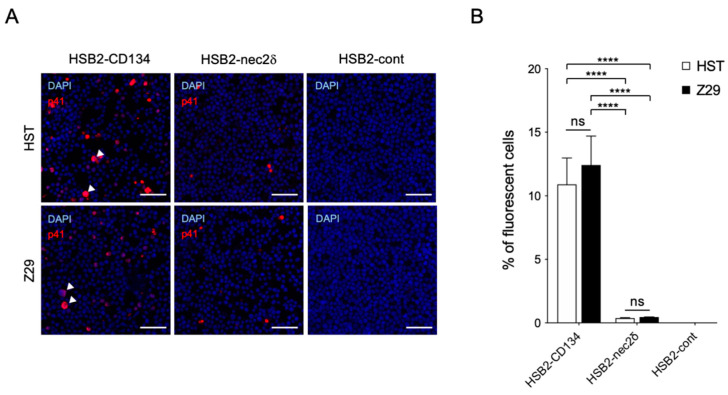
Infectivity of HST and Z29 in HSB2-nec2δ and HSB2-CD134 cells. (**A**) Viral antigen detection in HSB2-nec2δ, HSB2-CD134, and HSB2-cont cells determined using an immunofluorescence assay. Cells were infected with HHV-6B HST and Z29 strains (multiplicity of infection = 0.1) and then treated with cold acetone at 48 h post-infection. HHV-6 p41 protein (red) was detected in virus-infected HSB2-nec2δ and HSB2-CD134 cells. HSB2-cont cells were used as a negative control. Nuclei of cells were stained with DAPI (blue). Arrowhead indicates balloon-like cells. Scale bars represent 50 μm. (**B**) Virus-infected cells (red) were counted. Error bars represent standard deviations (*n* = 3 per cell). Asterisks and ns indicate statistical significance (**** *p* < 0.0001, Tukey’s multiple comparison test) and no significance, respectively.

We examined whether the susceptibility of nectin-2 as a viral entry-mediated molecule was different between the HST and Z29 strains. Viral antigens were detected in both HST- and Z29-infected-HSB2-nec2δ cells without a cytopathic effect (CPE), whereas some virus-infected HSB2-CD134 cells showed typical CPE (balloon-like cells) (Figure 4A). There was no difference in the infectivity of the HSB2-CD134 and HSB2-nec2δ cells in the HST and Z29 strains (Figure 4B). Viral infectivity using nectin-2 as a viral entry-mediated molecule was approximately 29 and 27 times lower than that of CD134 for HST and Z29 infection (*p* < 0.0001), respectively (Figure 4B).

### 3.5. Nectin-2 Enables HHV-6B Replication in CCRF-HSB-2 Cells

**Figure 5:**
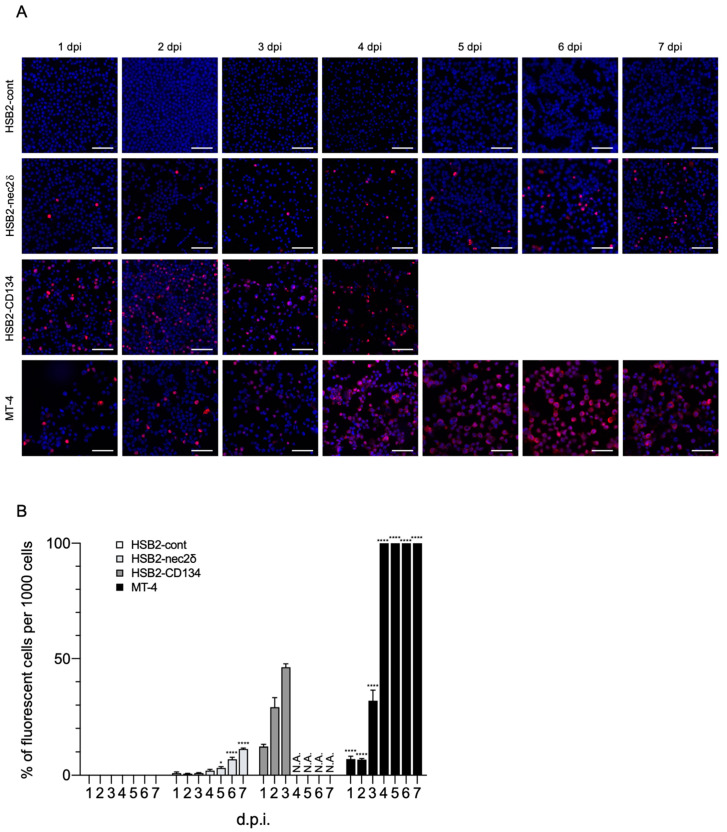
HHV-6B growth in HSB2-nec2δ cells. (**A**) Viral antigen detection in HSB2-cont, HSB2-nec2δ, HSB2-CD134, and MT-4 cells using an immunofluorescence assay. Cells were infected with the HHV-6B HST strain (multiplicity of infection = 0.1) and then treated with cold acetone for 1–7 days post-infection (d.p.i.). HHV-6 p41 protein (red) was detected in virus-infected HSB2-nec2δ and HSB2-CD134 cells. HSB2-cont cells were used as a negative control. HHV-6B-infected HSB2-CD134 cells died due to the lytic cycle at 4 d.p.i. Nuclei of cells were stained with DAPI (blue). Scale bars represent 50 μm. (**B**) Virus-infected cells (red) were counted 1–7 d.p.i. Error bars represent standard deviations (*n* = 3 per cell). Asterisks indicate statistical significances when compared with the HSB2-cont cells (* *p* < 0.05, **** *p* < 0.0001, Dunnett’s multiple comparison test except for the data of HSB2-CD134 because of missing values). N.A. indicates not applicable (uncountable due to lytic infection progression).

Viral replication was examined using HSB2-cont, HSB2-nec2δ, HSB2-CD134, and MT-4 cells for 7 days. Viral proteins were detected in HSB2-nec2δ, HSB2-CD134, and MT-4 cells (Figure 5A). The number of HHV-6B-infected HSB2-nec2δ cells increased gradually and reached a peak of 10% (Figure 5B). HHV-6B activity was low in HSB2-nec2δ cells until the fourth day and then increased, indicating that the infectious virus had been produced. The number of HHV-6B-infected HSB2-CD134 cells increased rapidly for 3 days and then these cells died via the lytic cycle 4 days post-infection (d.p.i.), whereas 100% of the MT-4 cells were infected by multi-step growth at 4 d.p.i. MT-4 cells are human T-cell lymphotropic virus type 1 (HTLV-1) which transform and express HTLV-1 Tax, which is a transcriptional activator of the viral long terminal repeat [49,50]. Tax might protect HTLV-1-infected T-cells from apoptosis [50]. Unlike HSB2-CD134 cells, MT-4 cells that are highly infected with HHV-6B might remain alive because of Tax.

### 3.6. Nectin-2 Interacts with HHV-6B gB

**Figure 6:**
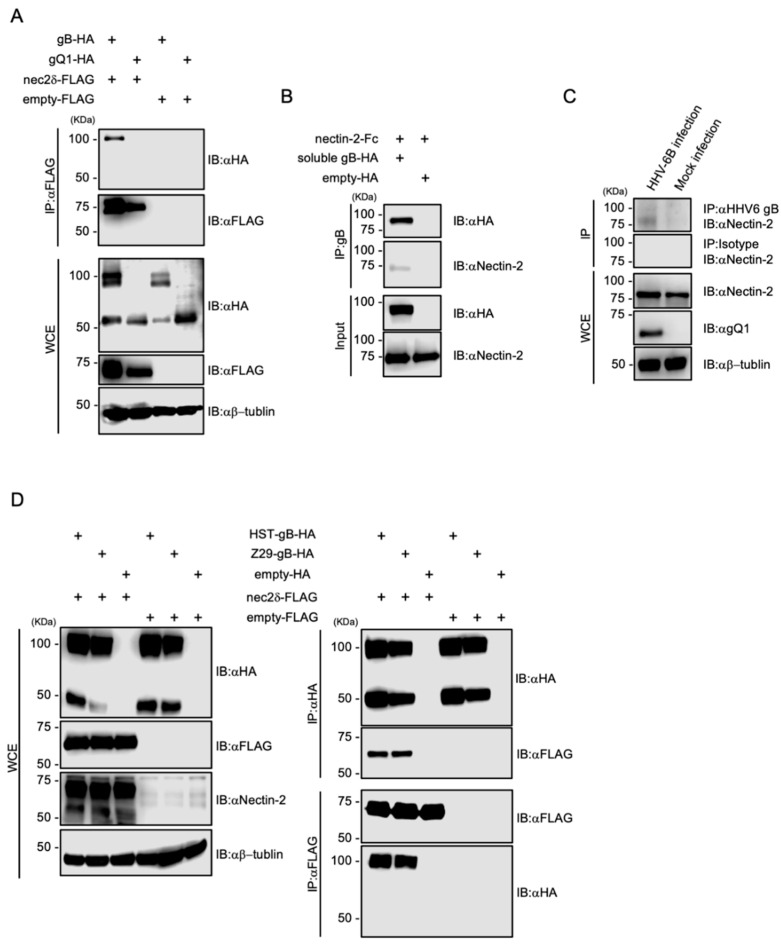
Interaction of HHV-6B gB with nectin-2. (**A**) The 293T cells were co-transfected with pCAGGS-HST-gB-HA or pCAGGS-HST-gQ1-HA and pCAGGS-nec2δ-FLAG or pCAGGS-FLAG. Cell lysates were subjected to immunoblotting (IB) and immunoprecipitation (IP) with the indicated antibodies. HHV-6B gB consists of three polypeptides of 102 kDa, and its proteolytic cleavage products of 59 and 50 kDa [52]. C-terminal HA-tagged recombinant gB proteins were expressed and used. (**B**) Recombinant nectin-2-Fc was immunoprecipitated with soluble HST-gB-HA or HA. (**C**) HHV-6B-infected MT-4 cells were cultured for 6 d and lysates were subjected to co-IP with anti-HHV-6 gB antibody (6A5). Mock-infected MT-4 cells were used as a negative control. (**D**) 293T cells were co-transfected with pCAGGS-gB (HST or Z29)-HA or pCAGGS-HA and pCAGGS-nec2δ-FLAG or pCAGGS-FLAG. Cell lysates were subjected to IB with the indicated antibodies (left panel). Cell lysates were subjected to IP with anti-FLAG or anti-HA antibodies (right panel). WCE, whole-cell extract.

To determine the ligand for nectin-2, we investigated the HHV-6B glycoproteins. Four envelope glycoproteins, gB, gD, gH, and gL, are essential for viral entry [18], and the gD of some alphaherpesviruses binds to nectin-2 [20,46,47]. HHV-6B contains at least eight envelope glycoproteins; however, there is no gD that is conserved among several alphaherpesviruses [26]. We focused on gB, as it is a fusion glycoprotein that is highly conserved among herpesviruses [51], and its cell surface receptors have not been identified in HHV-6B. We constructed HA-tagged HST gB and gQ1 as well as FLAG-tagged nectin-2 expression plasmids to evaluate the interactions between HHV-6B glycoproteins and nectin-2. Then, 293T cells overexpressing glycoproteins and nectin-2 were examined by co-IP. As a result, gB-HA and nectin-2-FLAG were immunoprecipitated using anti-FLAG and anti-HA antibodies (Figure 6A). Next, to confirm the direct binding of nectin-2 to HST gB under cell-free conditions, soluble HST gB-HA lacking a transmembrane domain was generated and examined by IP. Recombinant nectin-2 ectodomain tagged with the Fc portion of human IgG1 (nectin-2-FC) was immunoprecipitated with soluble HST gB-HA (Figure 6B). The bands at which the magnetic agarose bound to the Fc proteins were not detected. We also confirmed that nectin-2 interacts with gB in HHV-6B-infected cells. Co-IP was performed in HHV-6B-infected MT-4 cells, and nectin-2 was immunoprecipitated using gB (Figure 6C). To further address the nectin-2 interactions with gB, we constructed HA-tagged Z29 gB expression plasmids and evaluated the interactions between strain-specific gB and nectin-2. Both strains of gB-HA and nectin-2-FLAG were immunoprecipitated with each other (Figure 6D). This result is supported by the viral infectivity data for the HSB2-nec2δ cells between the HST and Z29 strains (Figure 4B).

Together, these findings indicate that nectin-2 interacts with HHV-6B gB.

### 3.7. The V-set Domain of Nectin-2 Is Important for Interactions with HHV-6B gB

**Figure 7:**
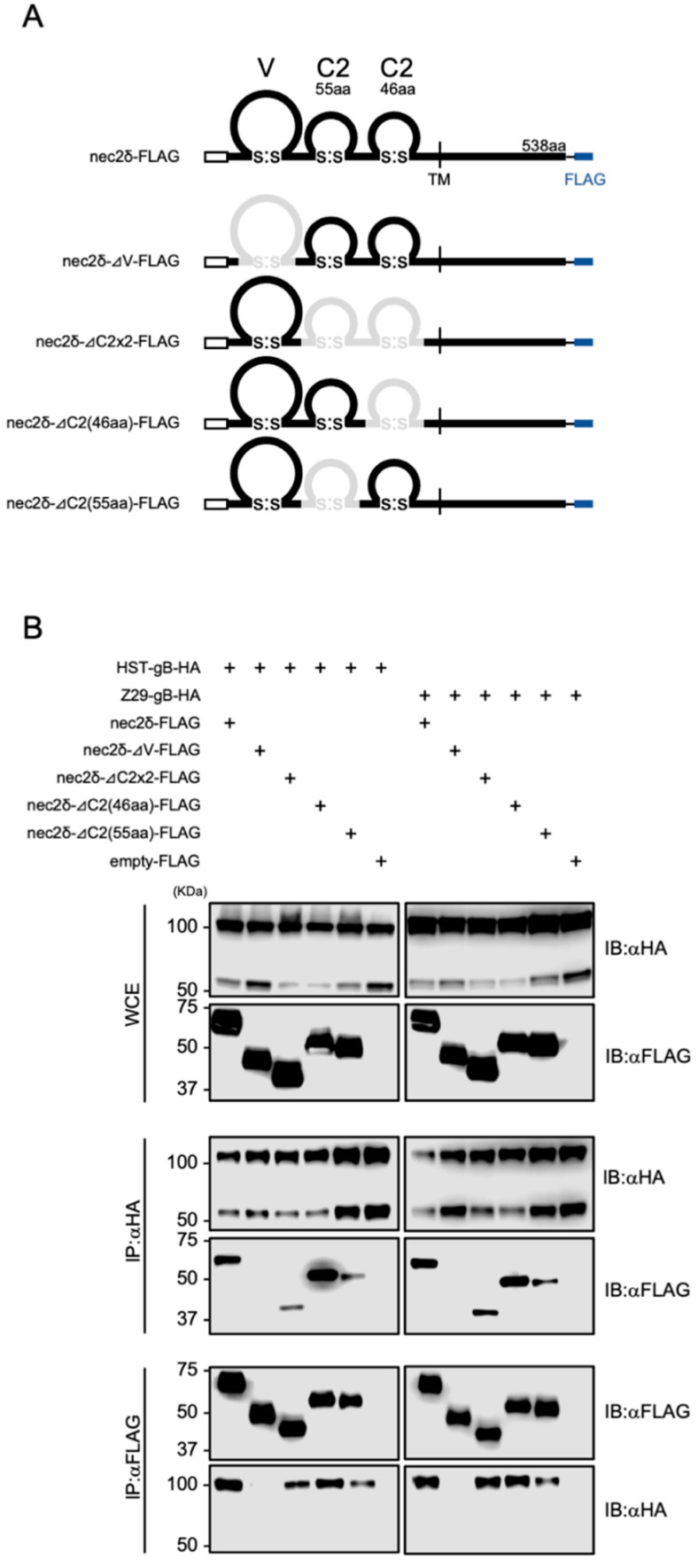
Interactions between HHV-6B gB and nectin-2 deletion mutants. (**A**) Schematic representation of the constructed nectin-2δ deletion mutants. Deleted domains are represented by gray lines. (**B**) 293T cells were transfected with plasmids encoding HHV-6B gB-HA and nectin-2δ-FLAG mutants and subjected to immunoblotting (IB) and immunoprecipitation (IP) with anti-FLAG or anti-HA antibodies. WCE, whole-cell extract.

To determine which domain of nectin-2 was required for binding to HHV-6B gB, several deletion mutants of nectin-2δ were constructed (Figure 7A). These plasmids were transfected into 293T cells together with the HHV-6B gB-expressing plasmids and examined by co-IP. The full-length domain, as well as the mutants lacking C2-set domains (ΔC2×2-FLAG), first C2-set domain (ΔC2(55aa)-FLAG), and second C2-set domain (ΔC2(46aa)-FLAG), but not a mutant lacking the V-set domain (ΔV-FLAG), were immunoprecipitated with HHV-6B HST and Z29 gBs (Figure 7B). These data demonstrate that the V-set domain of nectin-2 is important for interactions with HHV-6B gB.

## 4. Discussion

The HHV-6B entry receptor has previously been identified as CD134 [7], but the virus has been detected in tissues in which CD134 is not expressed [10,12,13,14]. Single receptors for the cell entry of HHV-6A, HHV-6B, and HHV-7 have been identified (CD46, CD134, and CD4, respectively) [7,53,54]. While single receptors have been identified for the entry of HHV-6A (CD46), HHV-6B (CD134), and HHV-7 (CD4) into T cells, other herpesviruses use multiple receptors that enable entry into diverse cell types [18]. gB, gH, gL, gM, and gN are conserved glycoproteins from the family *Herpesviridae* [55,56]. Among them, gB and gH/gL play key roles in membrane fusion and herpesvirus infection [57], and the gB and gH of HHV-6 also induce cell-to-cell fusion [58]. In HHV-6B, gH is a component of a tetrameric complex (i.e., gH/gL/gQ1/gQ2) [59], which is a viral ligand for the receptor CD134 [7]. The binding of a viral receptor to gB has not been reported for HHV-6B. In this study, we demonstrated that the ectopic expression of nectin-2 conferred HHV-6B susceptibility to cells normally insensitive to the virus and that the infectivity of HHV-6B was reduced in nectin-2 knockout salivary gland cells. Furthermore, we demonstrated that nectin-2 interacts with HHV-6B gB.

Nectins are a family of Ca2^+^-independent Ig-like CAMs, and there are four members, nectin-1 to nectin-4 [45]. All members, except for the nectin-1 splice variant (nectin-1γ), have an extracellular region containing three Ig-like domains (a distal V-set domain and two C-set domains), a single transmembrane region, and a cytoplasmic region. These molecules homophilically and heterophilically interact in *trans* with each other to form cell–cell adherens junctions (AJs) cooperatively with E-cadherin in a variety of cells [60]. Among them, nectin-1 and nectin-2 have been identified as alphaherpesvirus (i.e., HSV-1, HSV-2, and PRV) entry receptors that interact with their respective viral gDs [19,20]. Interestingly, our data showed that HHV-6B gB, which has no known sequence or structural similarities with alphaherpesvirus gDs, interacts with nectin-2 (Figure 6, Figure 7 and Figure S3).

Ig-like molecules are used as entry receptors for alphaherpesviruses, as well as other viruses (e.g., nectin-4 and CD150 for measles virus, CD4 for human immunodeficiency virus, and CD155 for poliovirus) [61,62,63,64]. Notably, the V domain of these molecules is critical for virus entry into the cell [47,65,66,67,68,69]. In HSV-1, gD binds to nectin-1, and gD contacts the β-sheet of the V-set domain [70,71]. Martinez et al. [47] reported that the V-set domain of nectin-2 was critical for HSV entry via this molecule. Although the amino acid identity in the V-set domain between nectin-1 and nectin-2 is approximately 67% [46], the overall conformation of the nectin-2 V-set domain is similar to that of nectin-1 [72]. Similarly, the CD155 V-set domain has also been identified as an important binding site for poliovirus [69,73]. It seems that the equivalent region of the V domain of different Ig molecules can be utilized by several viruses for binding to the cell surface and for entry [47]. A similar region within the V-set domain was predicted to be the HHV-6B gB-binding site in nectin-2.

Although HSB2-cont cells and parent CCRF-HSB-2 cells expressed nectin-2 that was detectable using FACS (Figure 3), they did not permit viral replication, possibly because of the low affinity of the interaction. Indeed, recombinant nectin-2-Fc was found to be weakly bound to gB-HA in a nectin-2 direct binding assay (Figure 6B), and the efficiency of viral infection was low, even in the ex6 C7 cells, in which the mean fluorescence intensity was approximately three times higher than that in the HSB2-cont cells (Figure 3C,E). Therefore, nectin-2 must be overexpressed to support effective viral entry.

HHV-6B has been detected in T-cells, salivary glands, liver cells, and neural cells, which do not express CD134 [10,12,13,74]. In this study, we identified nectin-2 as an HHV-6B entry-mediated molecule using a cDNA library derived from a parotid gland cell line, HSY. Although nectin-2 might not be an entry receptor comparable to CD134, induction of virus infection via the overexpression of nectin-2 suggests that nectin-2 is involved in binding as well as the entry process. Nectin-2 is ubiquitously expressed and localized in AJs in a variety of cells and salivary glands [45]. In polarized epithelial cells (apico-basal cell polarity), intercellular adhesion is mediated through a junctional complex that comprises tight junctions (TJs), AJs, and desmosomes (DSs), and TJs are formed at the apical side of the AJs [75]. TJs provide a physical barrier that prevents the lateral movement of proteins between the apical and basolateral membranes [76]. The functional significance of TJs in the salivary gland epithelium is similar (e.g., polarized saliva secretion and barrier maintenance between the extracellular environment and the glandular lumen) [76]. We hypothesize that HHV-6B infection of salivary glands in vivo can be explained by two molecules, nectin-2 and CD134. HHV-6B infects T-lymphocytes via CD134-mediated entry, and infected T cells transfer HHV-6B to epithelial cells via the nectin-2 expressed at AJs in the salivary glands through the basolateral side. In the epithelia, viruses (transferred and/or propagated) are released from the apical region to the luminal side of the salivary glands. This mechanism is similar to that through which the measles virus infects the epithelia, which is mediated by nectin-4, and virus particles released from the apical membrane enter the luminal side of the respiratory tract [77]. It can be speculated that the number of HHV-6B-infected cells might be decreased at the time of entry into the salivary gland cells via nectin-2, and progeny viruses might be less productive in salivary glands. Further studies are required to improve our understanding of why nectin-2 represents low-efficiency HHV-6B entry and how nectin-2 mediates virus entry into the salivary glands.

In this study, we found that HHV-6B mediates nectin-2 and its entry into salivary gland cells (intercalated duct cells of the parotid gland). As nectin-2 is ubiquitously expressed in a variety of cells, including hepatocytes and astrocytes [16,75,78], this molecule may play a key role in the pathogenesis of HHV-6B-associated diseases in the liver and central nervous system. Further studies of these interactions will provide new insights into HHV-6B infection.

## Supplementary Materials

The following are available online at https://www.mdpi.com/article/10.3390/v14010160/s1, Figure S1: Target sequences in the nectin-2 knockout ex6 C7 cell clones, Figure S2: CD134 without the transmembrane anchor sequence was not detected inside the ex6 C7 cells, Figure S3: Comparison of the gD and gB amino acid sequences.
