# Supplementary material for: Nectin-2 Acts as a Viral Entry Mediated Molecule That Binds to Human Herpesvirus 6B Glycoprotein B
Source: Viruses. 2022 Jan 16;14(1):160. doi: 10.3390/v14010160 (PMC8779676; doi:10.3390/v14010160)
Supplement: Supplementary file 1 [file viruses-14-00160-s001.zip › viruses-1527835-supplementary.pdf]

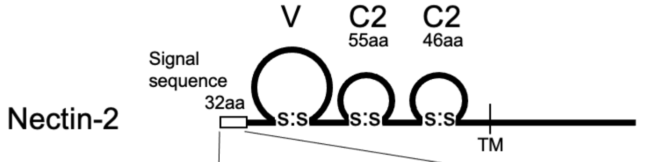

Nucleotide sequences

```
ex6 C7           1 ATGGCCCGGGCCGCTGCCCTCCTGCCGTCG-AGATCGCCGCCGACGCCGCTGCTGTGGCCGCTGCTGCTGCTGCTCCTGGAAACCG 88
ex6 C7 nec2-K01  1 ..............................--...................................................... 87
ex6 C7 nec2-K02  1 ..............................A....................................................... 89
ex6 C7 nec2-K03  1 ..............................A....................................................... 89
```

Amino acid sequences

```
ex6 C7           1 MARAAALLPSRSPPTPLLWPLLLLLLLET 29
ex6 C7 nec2-K01  1 MARAAALLPSDRRRRRCCGRCCCCCSWKP 29
ex6 C7 nec2-K02  1 MARAAALLPSKIAADAAAVAAAAAAAPGN 29
ex6 C7 nec2-K03  1 MARAAALLPSKIAADAAAVAAAAAAAPGN 29
```

Figure S1. Target sequences in the nectin-2 knockout ex6 C7 cell clones. Schematic representation of the nectin-2, the edited nucleotide sequences, and the resulting amino acid sequences. Sequence analysis showed that the nectin-2 gene of ex6 C7-nec2-KO1 cells had a 2-nucleotide deletion in the signal sequence and that the ex6 C7-nec2-KO2 and-KO3 cells had a 1-nucleotide insertion, leading to the abolishment of a signal peptide and nectin-2 expression.

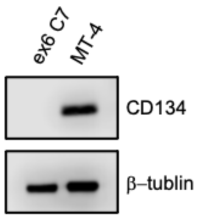

Figure S2. CD134 without the transmembrane anchor sequence was not detected inside the ex6 C7 cells. Ex6 C7 cells (7.5×10^5 cells) were washed with cold PBS, and then treated with 50 μL of cold lysis buffer containing a protease inhibitor cocktail for 15 min at 4°C. Cell lysates were sonicated at 4°C for 10 s and centrifuged at 21,000 × g for 10 min at 4°C. Aliquots of the supernatants (soluble fraction) were used for immunoblotting with anti-human CD134 monoclonal antibody (mAb) (clone: Ber-ACT35) and anti-β-tubulin mAb. MT-4 cells were used as the positive control.

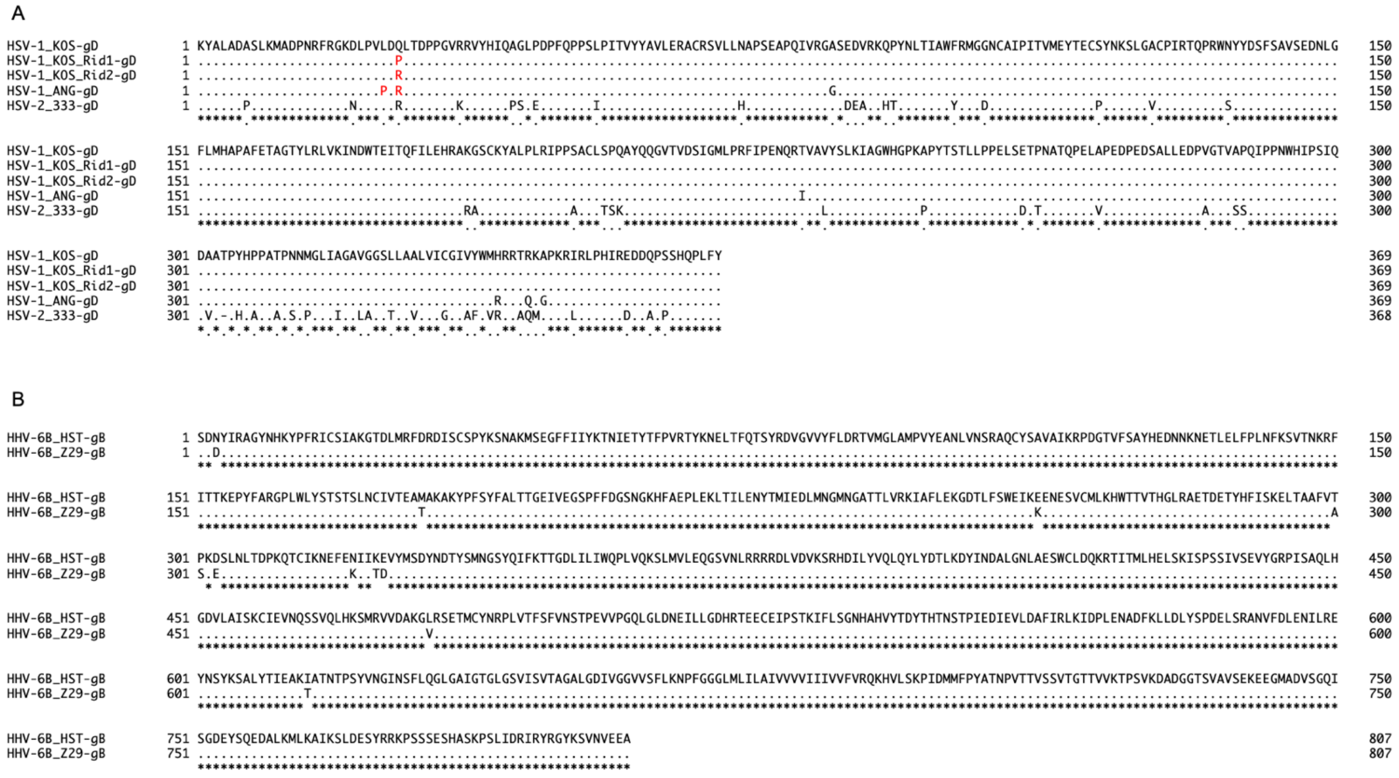

Figure S3. Comparison of the gD and gB amino acid sequences. (A) gD amino acid sequences in the HSV-1 strain KOS, HSV-1 mutant strains, and HSV-2 strain 333. The 25-amino acid signal sequence is excluded. The amino acid substitutions (L25P, Q27P, and Q27R) in HSV-1 that could be important for entry in nectin-2 expressing cells are identified in red (Dean *et al*., 1994. *Virology*, Lopez *et al*., 2000. *J Virol*). (B) gB amino acid sequences in HHV-6B strains HST and Z29. The 31-amino acid signal sequence is excluded.
